# Extensive frontal focused ultrasound mediated blood–brain barrier opening for the treatment of Alzheimer’s disease: a proof-of-concept study

**DOI:** 10.1186/s40035-021-00269-8

**Published:** 2021-11-05

**Authors:** So Hee Park, Kyoungwon Baik, Seun Jeon, Won Seok Chang, Byoung Seok Ye, Jin Woo Chang

**Affiliations:** 1 Brain Research Institute, Department of Neurosurgery, Yonsei University College of Medicine, Seoul, Korea; 2 Department of Neurology, Yonsei University College of Medicine, Seoul, Korea

**Keywords:** Alzheimer disease, Focused ultrasound, Blood–brain barrier, Amyloid beta-peptides

## Abstract

**Background:**

Focused ultrasound (FUS)-mediated blood–brain barrier (BBB) opening has shown efficacy in removal of amyloid plaque and improvement of cognitive functions in preclinical studies, but this is rarely reported in clinical studies. This study was conducted to evaluate the safety, feasibility and potential benefits of repeated extensive BBB opening.

**Methods:**

In this open-label, prospective study, six patients with Alzheimer’s disease (AD) were enrolled at Severance Hospital in Korea between August 2020 and September 2020. Five of them completed the study. FUS-mediated BBB opening, targeting the bilateral frontal lobe regions over 20 cm^3^, was performed twice at three-month intervals. Magnetic resonance imaging, ^18^F-Florbetaben (FBB) positron emission tomography, Caregiver-Administered Neuropsychiatric Inventory (CGA-NPI) and comprehensive neuropsychological tests were performed before and after the procedures.

**Results:**

FUS targeted a mean volume of 21.1 ± 2.7 cm^3^ and BBB opening was confirmed at 95.7% ± 9.4% of the targeted volume. The frontal-to-other cortical region FBB standardized uptake value ratio at 3 months after the procedure showed a slight decrease, which was statistically significant, compared to the pre-procedure value (− 1.6%, 0.986 *vs*1.002, *P* = 0.043). The CGA-NPI score at 2 weeks after the second procedure significantly decreased compared to baseline (2.2 ± 3.0 *vs* 8.6 ± 6.0, *P* = 0.042), but recovered after 3 months (5.2 ± 5.8 *vs* 8.6 ± 6.0, *P* = 0.89). No adverse effects were observed.

**Conclusions:**

The repeated and extensive BBB opening in the frontal lobe is safe and feasible for patients with AD. In addition, the BBB opening is potentially beneficial for amyloid removal in AD patients.

**Supplementary Information:**

The online version contains supplementary material available at 10.1186/s40035-021-00269-8.

## Background

Alzheimer’s disease (AD) is the most common cause of dementia and is characterized by progressive memory decline. The pathological hallmarks of AD are extracellular amyloid-beta (Aβ) plaques and intracellular neurofibrillary tangles [1]. Since the discovery of Aβ in 1984, Aβ accumulation has been considered the initial event in the AD process, and many anti-amyloid trials have been conducted in AD patients [2]. However, so far no therapies have been found to delay the progression of cognitive and functional disability.

The failure of prior anti-amyloid trials has stimulated investigation of alternative treatment approaches [3]. One of the challenges to anti-amyloid trial is the determination of effective dosing protocols that enable anti-amyloid antibody to penetrate the blood–brain barrier (BBB) effectively without undesirable side effects such as amyloid-related imaging abnormalities. As such, focused ultrasound (FUS) with microbubble-mediated temporary BBB opening has been attracting attention for its role in improving the delivery of therapeutic agents to the brain [4].

In addition, BBB opening alone has an anti-amyloid effect. Several preclinical studies have demonstrated that the FUS-mediated BBB opening can reduce Aβ and phosphorylated tau burden and improve cognitive function [5–8]. Based on these results, two clinical trials of BBB opening for AD have been undertaken: the phase 1 clinical trial by Lipsman et al. targeting the right frontal lobe [9], and the phase 2 clinical trial by Rezai et al. targeting the hippocampus and entorhinal cortex [10]. The Lipsman’s study demonstrates for the first time the safety of BBB opening in humans, while Rezai et al. demonstrate an Aβ decrease after BBB opening [11]. However, neither study showed any significant improvement in cognitive impairment. In addition, several other clinical trials are currently ongoing or completed: the BBB opening trial using single-element FUS for early AD or mild cognitive impairment patients (NCT04118764) and BBB opening trial using an implanted FUS device for mild AD (NCT03119961).

Since Aβ deposition and subsequent changes widely occur throughout the brain and previous studies have reported a small-range opening of less than 3 cm^3^, in this study we aimed to evaluate extensive BBB opening of above 20 cm^3^. As the safety of BBB opening in very large areas has not yet been confirmed, in order to avoid serious potential complications from BBB opening of the mesial temporal area, we selected the prefrontal area which also has much accumulation of Aβ. The potential secondary benefits, such as cerebral Aβ removal or clinical improvement, which have been found in preclinical studies [5–8], were also assessed.

## Methods

### Study design

**Fig. 1:**
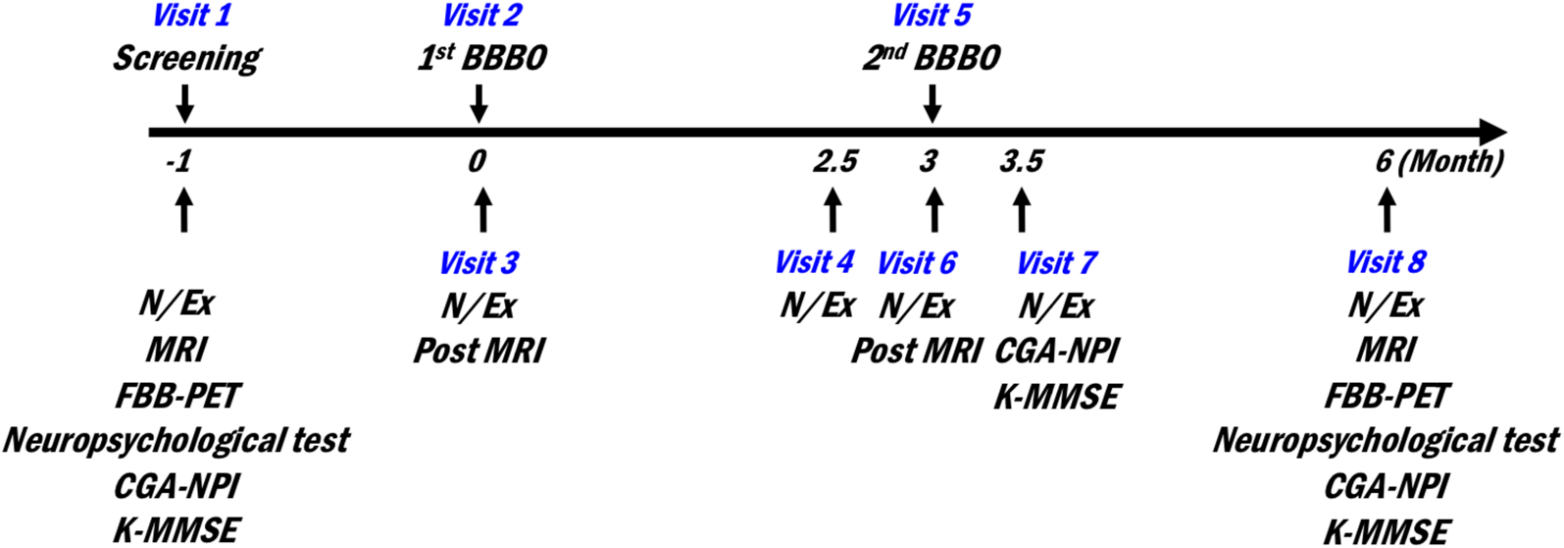
Overview of the study design. BBBO, BBB opening; N/Ex, Neurological examination; MRI, magnetic-resonance imaging; FBB-PET, ^18^F-Florbetaben positron emission tomography; CGA-NPI, Caregiver-Administered Neuropsychiatric inventory; K-MMSE, Korean Version of Mini-mental State Examination

This study was an open-label, prospective study designed to evaluate the safety, feasibility and efficacy of repeated and extensive BBB opening of the bilateral frontal lobes. FUS-mediated BBB opening was performed twice at three-month intervals. Before the procedures, magnetic resonance imaging (MRI), ^18^F-Florbetaben (FBB) positron emission tomography (PET) and comprehensive neuropsychological tests were performed. To assess the safety and feasibility, MRI was performed after each BBB opening procedure. To assess the efficacy, FBB PET and comprehensive neuropsychological tests were performed 3 months after the second procedure, a time point selected based on the concerns of learning effect due to repeated tests within a short period of time. Instead, Korean version of Mini-Mental State Examination (K-MMSE) and Caregiver-Administered Neuropsychiatric Inventory (CGA-NPI), which obtain patients' symptom information from their caregivers, were performed 2 weeks after the second procedure. The outline of study design is shown in Fig. 1.

### Participants

Participants were enrolled from AD patients who visited the neurology or neurosurgery outpatient clinic of our hospital. AD patients aged 50–85 and having a K-MMSE score of ≤ 23 were eligible for the study. All participants fulfilled the criteria for probable AD dementia with high levels of biomarker evidence, and were identified as having significant cerebral Aβ deposition on FBB PET and neuronal injury relevant for AD on ^18^F-fluorodeoxyglucose PET scans [12]. Those with contraindications to BBB opening with microbubble-enhanced focused ultrasound were excluded. Detailed inclusion and exclusion criteria are listed in Additional file 1: Table S1. During the study period, the patients’ medications were maintained at the same dose.

### Standard protocol approvals, registrations, and patient consents

The study was approved by the Korean Food and Drug Administration and Institutional Review Board of our institution before study initiation (No. 1-2019-0095). Written informed consent was obtained from all participants and their primary caregivers. The clinical trial registration information can be found at http://www.clinicaltrials.gov/, identifier: NCT04526262.

### Magnetic resonance-guided FUS (MRgFUS) procedure

BBB opening was performed with the MRgFUS system (ExAblate Neuro Model 4000 Type 2.0 [220 kHz] system, InSightec, Haifa, Israel) under continuous infusion of a microbubble contrast agent (Definity®) (250 ml N/S + 1.3 ml Definity®, infusion rate 180 ml/h). Target regions were selected in the bilateral frontal lobes, mainly the prefrontal area, and they were set around the boundary between gray matter and white matter in both frontal lobes, avoiding sulci, vessel, and ventricle. As the height of the area to be sonicated once was about 7–9 mm, the targets were set at 1–1.5 cm intervals in height to avoid overlap. The inferior target was selected at the area 1 cm above the ventral aspect of the frontal lobe, and the middle and superior targets were above it by one and two intervals, respectively. BBB opening was performed in the maximum volume to set, taking into account the recommended protocol of microbubble contrast for FUS-mediated BBB opening. The BBB opening was started with a power of 8 W, which was gradually increased until the target accumulated cavitation dose reached 0.4–0.65, up to a maximum power of 40 W. Sonication was performed for 90 s per session. When inertial cavitation was confirmed, sonication was stopped immediately. In the case where a sufficient dose was not reached despite sufficiently high power, an additional 120-s sonication was used. After completion of the procedure, a gadolinium-enhanced MRI was performed to verify BBB opening.

### Primary outcome

The primary outcome was the overall clinical and radiological safety and the feasibility of repetitive extensive BBB opening in the bilateral frontal lobes. Clinical safety assessments included vital signs, adverse effects, neurological examination, blood tests, and electrocardiography. Radiological safety was evaluated based on the brain MRI to detect any sign of hemorrhage, edema or any other adverse radiological events. Brain MRI was performed immediately after each procedure and three months after the second procedure.

The feasibility of BBB opening was evaluated by comparing post-procedure gadolinium-enhanced MRI scans with pre-procedure MRI scans. The contrast-enhanced area was manually segmented by two neurosurgeons who were not involved in the procedure, based on the immediate post-procedure gadolinium-enhanced MRI, after which the target volumes were compared through linear image-based registrations on the pre-procedure MRI scans in which the targets were planned. Results from the two independent observers were averaged.

### Secondary outcome

#### Reduction of Aβ deposition

As previous studies have shown that cerebral Aβ accumulates even in the AD dementia stage [13], Aβ deposition was assessed with FBB PET using a Discovery 600 system (GE Healthcare, Milwaukee, WI) to identify the effect of localized FUS in the frontal lobe. FBB PET scans were carried out at screening (Visit 1) and 3 months after the second procedure (Visit 8). FBB PET images were linearly registered to the individual T1-weighted MRI scans at corresponding time point using rigid body transformation. Then, the reconstructed cortical surfaces and classified tissues from the CIVET processing pipeline (http://mcin.ca/civet) were linearly registered into the PET images by applying inverse transform matrices. We performed partial volume correction within gray and white matter regions using iterative deconvolution with a surface-based anatomically constructed filtering method that uses the representation of the volume between the inner and outer surfaces as a spatial constraint to the PET signal [14]. Then the corrected PET values were normalized to the cerebellar crus-I/II reference region, resulting in standardized uptake value ratios (SUVRs). Based on the Desikan-Killiany-Tourville (DKT) parcellation atlas, we extracted the global SUVR value as the cortical volume-weighted average of the following cortical regions of interest (ROI): frontal, anterior/posterior cingulate, parietal, and lateral temporal cortices. These ROIs are similar to previous studies using FBB PET for measurement of Aβ deposition, but the occipital ROI was excluded in our study because this ROI shows low Aβ load in AD-related changes [15]. We evaluated the entire frontal lobe including but not restricted to the BBB opening site. Specifically, the frontal ROI included the superior frontal, middle frontal, inferior frontal, and orbitofrontal cortices. We then calculated the SUVR of the frontal ROI to other cortical regions including the lateral temporal, lateral parietal, and anterior/posterior cingulate lobes. The lateral temporal ROI covers the middle temporal lobe and the superior temporal lobe. The parietal ROI covers the supramarginal gyrus, superior parietal lobe, precuneus, and inferior parietal lobe.

#### Changes in neuropsychological test scores and neuropsychiatric symptoms

All study participants underwent a detailed neuropsychological evaluation using the Seoul Neuropsychological Screening Battery [16] on Visit 1 and Visit 8. Standardized z scores were available for all scorable tests based on age- and education-matched norms. Among the scorable tests, we included the digit span backward test for the attention domain; the Korean version of the Boston Naming Test for the language domain; the copying item of the Rey–Osterrieth Complex Figure Test (RCFT) for the visuospatial domain; 20-min delayed recall item of the RCFT and Seoul Verbal Learning Test for the memory domain; and the phonemic Controlled Oral Word Association Test (COWAT), semantic COWAT, and the Stroop color reading test for the frontal/executive domain. Additionally, general cognition and daily functioning were assessed using the K-MMSE, Clinical Dementia Rating (CDR), CDR Sum of Boxes (CDR-SOB), and Korean Instrumental Activities of Daily Living (K-IADL) [17]. K-MMSE scores were additionally assessed at two weeks after the second procedure (Visit 7).

We also assessed patient’s neuropsychiatric symptoms from caregivers using the CGA-NPI [18] at Visit 1, Visit 7 and Visit 8. The CGA-NPI evaluated 12 behavioral and psychological symptoms of dementia including delusion, hallucinations, agitation/aggression, depression/dysphoria, anxiety, elation/euphoria, apathy/indifference, disinhibition, irritability/lability, aberrant motor behavior, sleep/nighttime behavior disorders, and appetite/eating change. Each of the symptoms was retrospectively rated for the four weeks prior to the interview.

### Statistical analysis

Statistical analyses for clinical data were performed with Statistical Package for the Social Sciences version 25.0 software (IBM Corp., Armonk, NY). Differences in clinical data between pre- and post-MRgFUS were tested using a non-parametric paired Wilcoxon signed-rank test. *P* < 0.05 was considered as statistically significant.

## Results

### Participants

**Fig. 2:**
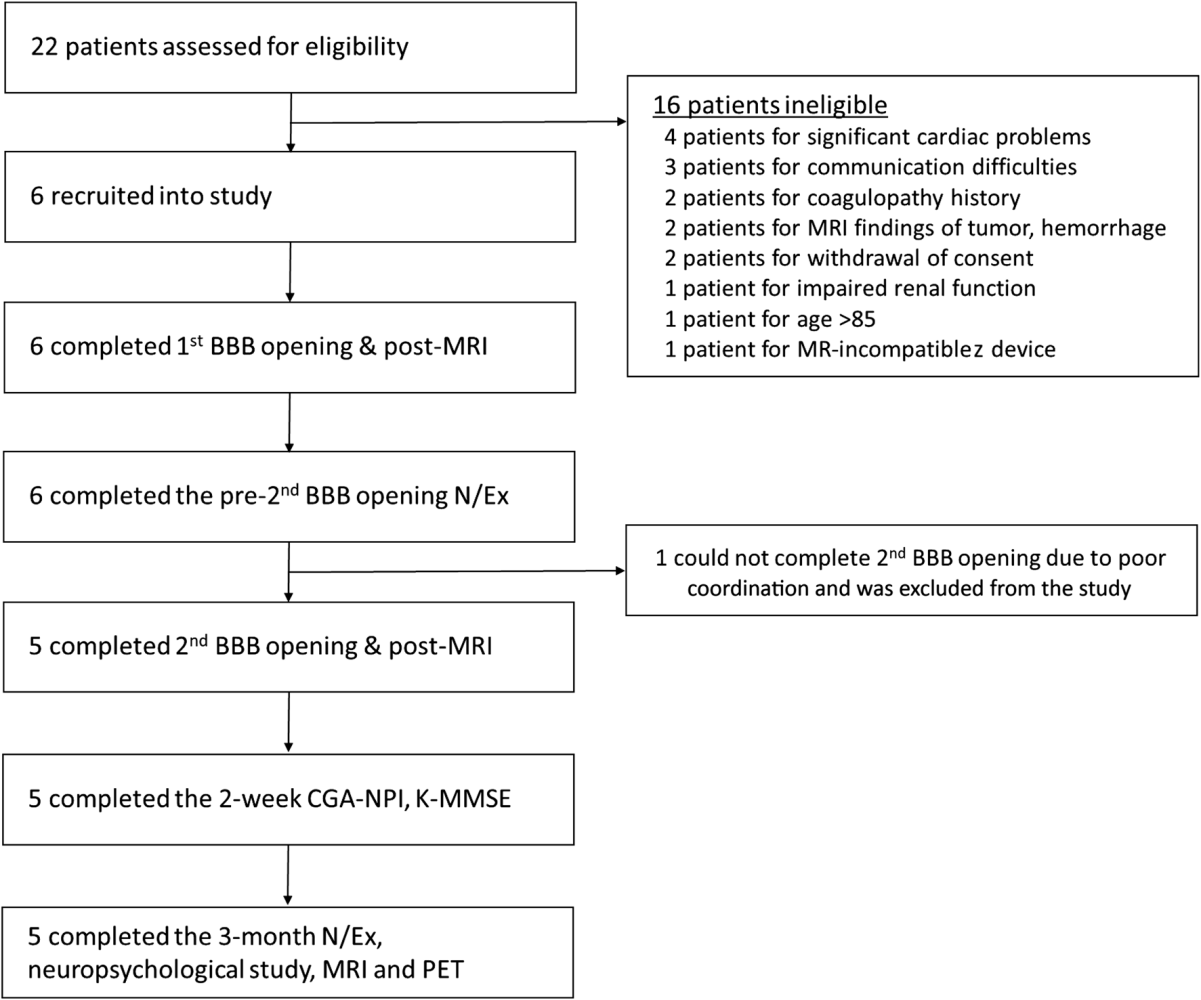
Flow chart of the study. BBB, Blood–brain barrier; N/Ex, Neurological examination; MRI, magnetic-resonance imaging; CGA-NPI, Caregiver-Administered Neuropsychiatric inventory; K-MMSE, Korean version of mini-mental state examination; PET, positron emission tomography

We enrolled five female and one male patients between August 2020 and September 2020. Five patients completed the two consecutive BBB opening procedures. One patient did not complete the second BBB opening procedure due to poor cooperation during MRI, and thus was excluded from analysis (Fig. 2). Demographics of the five included patients are shown in Table 1. The mean baseline K-MMSE score was 17.4 ± 5.2 and the mean baseline CDR-SOB score was 6.3 ± 3.1. The mean baseline FBB global SUVR was 1.937 ± 0.163.

**Table 1:** Patient demographics

	Age (years)	Sex	Education (years)	*ApoE4* carrier	Comorbidities	Medications
Case 1	58	F	6	Yes	None	Rivastigmine 18 mg, Choline alfoscerate 800 mg, Lexapro 5 mg
Case 2	80	F	9	Yes	None	Rivastigmine 18 mg, Choline alfoscerate 800 mg, Lexapro 5 mg
Case 3	55	F	12	Yes	Myasthenia gravis	Rivastigmine 27 mg, Memantine 5 mg, Lexapro 5 mg, Prednisolone 5 mg, Tacrolimus 3 mg
Case 4	85	F	12	Yes	Hypertension, osteoporosis	Donepezil 10 mg, Candesartan 16 mg, Amlodipine 5 mg, Calcium 500 mg, Cholecalciferol 1000 IU
Case 5	75	M	12	No	None	Galantamine 16 mg
Mean (SD)	70.6 (13.5)		10.2 (2.7)			

*SD* standard deviation

### Primary outcome

The five participants were all tolerable during procedures. No adverse events occurred during the entire study period, including in the case of the excluded sixth patient. Neither systemic nor neurological worsening was reported. Radiologically, there was no brain edema, overt cerebral hemorrhage or infarction during and after the study.

**Fig. 3:**
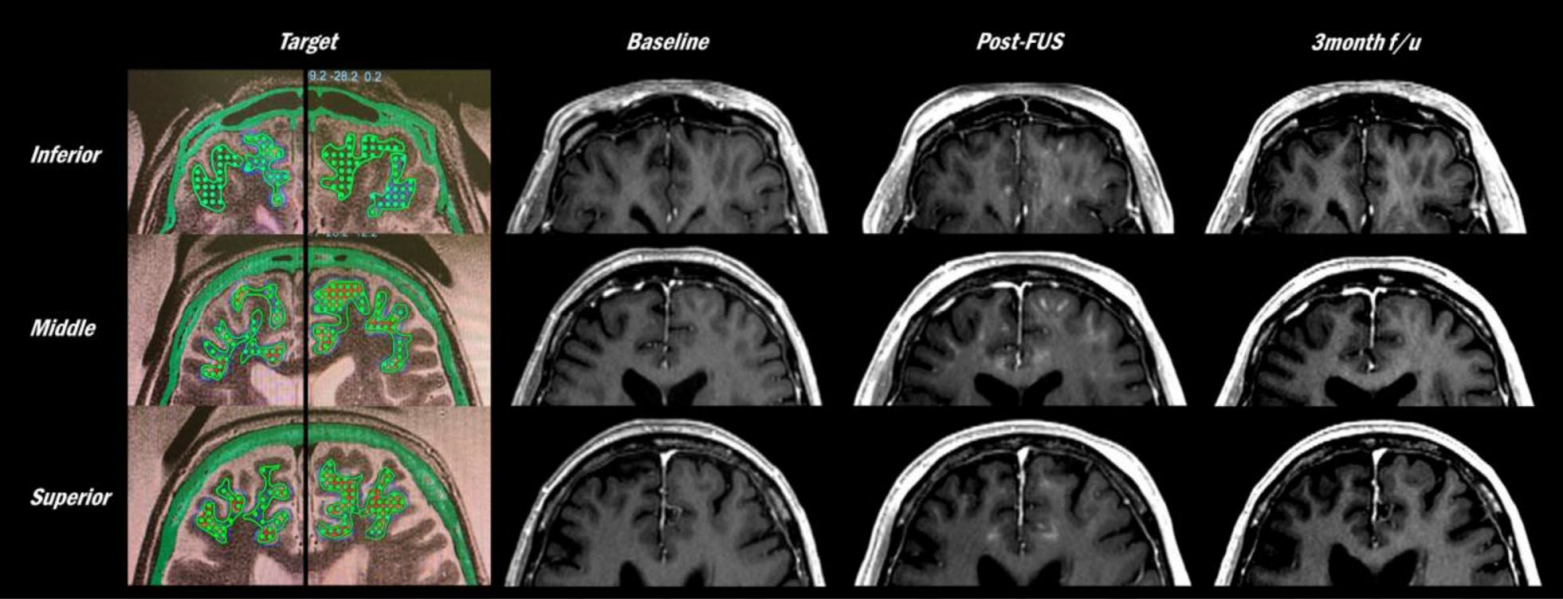
The targets and MRI demonstration of BBB opening and closure in Patient 3. Immediately post-FUS gadolinium-enhanced T1 MRI showed that parenchyme at target was well enhanced, indicating the successful BBB opening. Afterwards, this area did not show enhancement in MRI at 3 months post-procedure, indicating that the BBB was  temporarily opened and closed

We targeted a mean volume of 21.1 ± 2.7 cm^3^ in the bilateral frontal lobes (Table 2). Immediate post-procedure gadolinium-enhanced MRI scans showed that 95.7% ± 9.4% of the target was well enhanced (Fig. 3). MRI scans performed 3 months after the second procedure showed no enhancement in this area, indicating the closure of BBB.

**Table 2:** Feasibility of blood–brain barrier (BBB) opening as assessed by post-procedure MRI

	Volume (cm^3^)*	Ratio of BBB opening to target (%)^†^
Case 1	17.7	78.9
Case 2	20.0	100
Case 3	20.6	100
Case 4	22.6	97.6
Case 5	24.7	100
Mean (SD)	21.1 (2.7)	95.7 (9.4)

*An average volume of two sessions of BBB opening

^†^An average ratio of BBB opening area to target

### Secondary outcome

#### Reduction in Aβ deposition

**Fig. 4:**
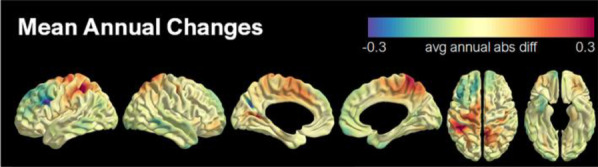
Mean annual changes in the ^18^F-Florbetaben uptake after treatment. The mean annual changes in the ^18^F-Florbetaben SUVR of five participants after treatment. The colors illustrate the average value of absolute annual differences for the ^18^F-Florbetaben SUVR between pre and post-treatment. SUVR, Standardized uptake value ratio

The frontal-to-other brain region FBB SUVR was significantly decreased at Visit 8 compared to Visit 1 (− 1.6%, 0.986 ± 0.065 *vs* 1.002 ± 0.063, *P* = 0.043) (Table 3). When the follow-up period of PET was annualized, the mean annualized changes in the global FBB SUVR for the five participants showed the greatest decline in the frontal lobe (Fig. 4, Additional file 1: Fig. S1).

**Table 3:** Global and regional ^18^F-Florbetaben SUVR

	Case 1		Case 2		Case 3		Case 4		Case 5		Mean (SD)		
	Visit 1	Visit 8	Visit 1	Visit 8	Visit 1	Visit 8	Visit 1	Visit 8	Visit 1	Visit 8	Visit 1	Visit 8	*P* value
Global SUVR	2.172	2.277 (+ 4.8%)	1.876	1.901 (+ 1.3%)	1.955	1.972 (+ 0.9%)	1.722	1.648 (− 4.3%)	1.959	1.941 (− 0.9%)	1.937 (0.163)	1.948 (0.224) (+ 0.6%)	0.686
Frontal SUVR	2.211	2.287 (+ 3.4%)	1.803	1.815 (+ 0.6%)	1.904	1.913 (+ 0.5%)	1.808	1.728 (− 4.4%)	1.955	1.896 (− 3.0%)	1.936 (0.167)	1.928 (0.214) (− 0.4%)	0.893
Frontal/Other SUVR	1.034	1.008 (− 2.5%)	0.935	0.925 (− 1.1%)	0.953	0.946 (− 0.7%)	1.091	1.089 (− 0.2%)	0.996	0.963 (− 3.3%)	1.002 (0.063)	0.986 (0.065) (− 1.6%)	0.043

Wilcoxon signed rank test was used for comparing Visit 1 (baseline) and Visit 8 (3 months post-FUS)

*SUVR* standardized uptake value ratio

#### Changes in neuropsychological test scores and neuropsychiatric symptoms

In neuropsychological test scores, K-IADL, and other tests performed at Visit 1 and Visit 8, there was no significant difference between visits. There were also no significant differences in K-MMSE score between Visit 1, Visit 7, and Visit 8 (Visit 1 *vs*  Visit 7, 17.4 ± 5.2 *vs*  17.0 ± 5.7, *P* = 0.58; Visit 1 *vs* Visit 8, 17.4 ± 5.2 *vs*  16.6 ± 5.1, *P* = 0.69). For neuropsychiatric symptoms, the total CGA-NPI score at Visit 7 was significantly decreased compared to that at Visit 1 (2.2 ± 3.0 vs. 8.0 ± 6.0, *P* = 0.042). However, the total CGA-NPI score at Visit 8 did not differ significantly from that at Visit 1 (5.2 ± 5.8 *vs*  8.0 ± 6.0, *P* = 0.89) (Table 4).

**Table 4:** Neuropsychological test scores and neuropsychiatric symptom test scores

	Case 1		Case 2		Case 3		Case 4		Case 5		Mean (SD)		*P* value
	Visit 1	Visit 8	Visit 1	Visit 8	Visit 1	Visit 8	Visit 1	Visit 8	Visit 1	Visit 8	Visit 1	Visit 8	
*Neuropsychological test score*													
Digit span backward	− 4.14	− 4.14	0.53	0.53	− 0.35	− 1.23	− 1.98	− 0.60	− 1.00	− 0.88	− 1.39 (1.79)	− 1.26 (1.74)	0.59
K-BNT	− 3.38	− 3.84	− 0.81	− 0.68	− 2.80	− 2.59	− 1.61	− 0.81	− 1.32	− 1.94	− 1.98 (1.07)	− 1.97 (1.31)	0.89
RCFT copy	− 7.20	− 7.48	− 1.88	− 2.00	− 9.14	− 9.37	0.93	1.00	− 1.23	− 0.94	− 3.70 (4.26)	− 3.76 (4.44)	0.69
SVLT delayed recall	− 2.97	− 2.97	− 2.30	− 2.30	− 3.40	− 3.40	− 1.42	− 1.63	− 2.74	− 2.51	− 2.57 (0.75)	− 2.56 (0.67)	0.66
RCFT delayed recall	− 2.34	− 2.34	− 2.07	− 2.07	− 3.08	− 2.91	− 1.23	− 0.96	− 2.54	− 2.36	− 2.25 (0.68)	− 2.13 (0.72)	0.11
COWAT semantic	− 2.99	− 3.21	− 0.17	− 2.08	− 2.56	− 2.32	− 1.91	− 1.40	− 2.08	− 2.65	− 1.94 (1.08)	− 2.33 (0.67)	0.50
COWAT phonemic	− 2.53	− 2.53	− 0.09	− 0.82	− 1.39	− 1.06	− 1.03	− 0.15	− 0.69	− 2.09	− 1.15 (0.91)	− 1.33 (0.97)	0.72
Stroop color reading	− 4.89	− 4.77	− 0.57	− 1.58	− 4.56	− 4.43	− 0.54	− 0.42	− 2.77	− 3.75	− 2.67 (2.09)	− 2.99 (1.90)	0.68
CDR	2	2	1	1	1	1	0.5	0.5	0.5	1	1.0 (0.6)	1.1 (0.55)	0.32
CDR-SOB	10	10	5.5	5.5	9	9	3.5	3.5	3.5	6	6.3 (3.1)	6.8 (2.7)	0.32
K-IADL	1.67	1.78	1.4	1.3	1.8	1.8	0.5	0.5	0.7	1.33	1.21 (0.58)	1.34 (0.53)	0.29

	Case 1		Case 2		Case 3		Case 4		Case 5		Mean (SD)		*P* value*		*P* value^†^	
	K-MMSE	CGA-NPI	K-MMSE	CGA-NPI	K-MMSE	CGA-NPI	K-MMSE	CGA-NPI	K-MMSE	CGA-NPI	K-MMSE	CGA-NPI	K-MMSE	CGA-NPI	K-MMSE	CGA-NPI
*K-MMSE and CGA-NPI score*																
Visit 1	9	9	22	16	16	11	21	2	19	2	17.4 (5.2)	8.0 (6.0)				
Visit7 (post-FUS)	8	3	20	1	16	7	23	0	18	0	17.0 (5.7)	2.2 (3.0)	0 .58	0.042		
Visit8 (3 months post-FUS )	10	7	18	0	19	14	23	9	13	5	16.6 (5.1)	5.2 (5.8)			0.69	0.89

Wilcoxon signed rank tests were used to compare the difference between the neuropsychological test z scores, CDR, CDR-SOB, K-IADL, K-MMSE and CGA-NPI of Visit 1 and Visit 8. Also, we compared the K-MMSE and CGA-NPI scores of Visit 1 and Visit 7 with same test. *P* < 0.05 was considered significant. Visit 1: baseline. Visit 7: 2 weeks post-FUS. Visit 8: 3 months post-FUS

*CDR* clinical dementia rating scale, *CDR-SOB* CDR sum of boxes, *CGA-NPI* caregiver-administrated neuropsychiatric inventory, *COWAT* controlled oral word association test, *K-BNT* Korean version of the Boston naming test, *K-IADL* Korean instrumental activities of daily living, Rey-Osterrieth complex figure Test, *K-MMSE* Korean version of mini-mental state examination, *SD* standard deviation, *SVLT* Seoul verbal learning test

^*^P value: Comparison between Visit 1 and Visit 7

^†^*P* value: Comparison between Visit 1 and Visit 8

## Discussion

In this study, we tested the safety and feasibility of extensive BBB opening targeting the bilateral frontal lobes in AD patients. The major findings were: (1) repeated extensive BBB opening was safe and feasible with no serious side effects; (2) extensive BBB opening induced a decrease in Aβ accumulation in the frontal lobe targeted; and (3) neuropsychiatric symptoms were transiently relieved after BBB opening. Our results highlight the safety, feasibility, and possible efficacy of extensive BBB opening.

Previous human trials of BBB opening with MRgFUS have targeted a relatively small area: approximately 1 cm^3^ frontal BBB opening in AD patients [9], a 2–3 cm^3^ hippocampal BBB opening in AD patients [10], 3.5 cm^3^ BBB opening in amyotrophic lateral sclerosis patients [19] and 5–6 cm^3^ BBB opening in glioblastoma multiforme patients [20]. Our study is the first human trial of extensive BBB opening targeting an average volume of 21.1 cm^3^, which achieved BBB opening without any adverse events. The participants tolerated the procedures well and experienced no clinical or radiological side effects throughout the study. Moreover, despite the fact that cerebral vasculature of AD patients is fragile and vulnerable [21, 22], extensive BBB opening is tolerable, safe and reproducible in moderate-to-severe AD patients.

Another important point of our study was the relative Aβ decrease in the frontal lobe in which the BBB was opened. Previous preclinical studies have shown that Aβ burden is reduced by scanning ultrasound [23] or FUS alone in AD models [5]. However, previous human trials have resulted in divergent results. In the study of Lipsman et al., BBB opening at the white matter of frontal lobe did not result in a change in Aβ deposition one week after the BBB opening [9]. On contrary, in the study of D’Haese et al., hippocampal BBB opening induced a relative Aβ reduction in the BBB-opening area on PET one week after the BBB opening [11]. Here our results showed the Aβ-reducing effect of BBB opening in humans.

The course of change of Aβ deposition induced by BBB opening is not yet known. In our study, unlike the previous two studies, PET was performed three months after the last procedure due to regulations in our country which restrict the interval and frequency of use of PET isotopes. Our results showed Aβ decrease even at 3 months after BBB opening procedure. It is not clear whether the Aβ decrease seen in our study is the maintenance state of Aβ decrease caused by the BBB opening or the relative decrease state seen in the process of re-accumulation after Aβ reduction. However, it is meaningful to confirm that relative Aβ decrease is seen even at 3 months after the procedure in circumstance where the changes after BBB opening are not well known in AD patients.

In this study, there was no change in the K-MMSE and comprehensive neuropsychological test scores. However, there was a transient improvement in neuropsychiatric symptoms after extensive frontal BBB opening. Although there is currently no placebo-controlled trial assessing the CGA-NPI score in AD patients, the placebo-controlled trials using NPI score, which is highly correlated with the CGA-NPI score and has same scoring system as CGA-NPI, have shown that the maximal placebo response to neuropsychiatric symptoms in AD reaches at week 4 with an expected reduction of NPI score for 1.2 [24]. In our study, however, as the change in CGA-NPI score was 5.8 at Visit 7 (2 weeks after second BBB opening), which was greater than the previously expected placebo effect, it can be considered that there is a real transient improvement effect from BBB opening. Considering that the neuropsychiatric symptoms in AD are associated with the structural and metabolic changes in the frontal cortex and the disruption of the fronto-temporal-subcortical network [25], extensive BBB opening in the frontal cortex may be associated with improvement of the symptoms. The improvement found in our study, which was not seen in other studies, may be attributed to the BBB opening area, which was several times larger than that of other studies, or the location of BBB opening, as the frontal lobe has many connections with other areas.

Currently, the exact mechanisms underlying the temporary or sustained improvement after BBB opening remain unknown. Besides reduction of Aβ [26], several other mechanisms have been suggested in previous preclinical studies, including inflammation-induced neurogenesis [27], delivery of endogenous antibodies [28], and modulation of the glymphatic [29] or cerebrospinal fluid clearance system [27]. The neuromodulatory effect may also be involved. A previous functional MRI study has shown that MRgFUS targeting the right frontal lobe can induce transient changes in the frontoparietal network [30].

However, in this study, the improvements in neuropsychiatric symptoms did not persist till three months after the second procedure. Although the closing timeline varies widely across parameters, FUS systems, and detection approaches, the BBB opening itself is temporary and usually restored after 4 h [31], and the duration of the effect of BBB opening in AD models is reported from 3 days to 2 weeks [32–34] in preclinical studies. However, how long the effect of BBB opening lasts in humans has not been studied. Previous clinical studies were conducted at intervals of 2 weeks or 1 month, whereas our study was conducted at 3-month intervals, which were longer than previously used. As a result, we were able to confirm that the effect of BBB opening could last for 2–4 weeks, after which the symptoms worsen again.

Although the clinical improvements did not last for several months in most patients of our study, this study demonstrated that BBB opening alone can improve symptoms in humans, consistent with previous preclinical studies. Future studies with repeated extensive BBB opening combined with adjuvant therapeutics, such as stem cell therapy, neurotrophic factors, or immunomodulatory drugs may show potentials, based on preclinical evidence that the repetitive procedure of FUS in combination with antibodies or other drugs results in better outcomes than FUS treatment alone [5, 6, 32]. Also, considering that several therapeutics for AD have failed due to the limited BBB permeability [35], MRgFUS, which is demonstrated to be safe, can be used to overcome the problem of limited BBB permeability.

This study has several limitations. First, our study had a small sample size. Although this study demonstrated clinical and radiographical safety of and possible clinical benefits from the MRgFUS-induced extensive BBB opening, these results are limited in their generalizability as such. In addition, the participants in our study were patients with moderate to severe AD and most of them were *APOE4* carriers (four of the five). It is necessary to interpret our results cautiously. Second, this study did not demonstrate objective cognitive improvements. The transient clinical benefits induced by extensive BBB opening could not be excluded as the neuropsychological evaluation was not performed at two weeks after the second MRgFUS procedure unlike CGA-NPI. The lack of cognitive improvements may also be related to the small sample size in this study and the study design. The open-label design of the current study prevented the inclusion of an AD control group without a MRgFUS procedure due to ethical concerns. As cognitive function deteriorates with the progression of AD, this could have limited the power of our study. In future studies, an AD control group matched for demographic factors and disease severity should be included. Third, there was no placebo-controlled arm in this study, so we cannot exclude the contribution of placebo effect to the transient improvement of CGA-NPI score. Although according to previous studies the placebo effect might not be the only factor in the transient improvement of neuropsychiatric symptoms in this study, a study with a placebo arm is also needed in the future. Fourth, the PET analysis in this study analyzed SUVR changes in the entire frontal lobe rather than specifically analyzing the volumes of brain regions subjected to BBB opening.

## Conclusions

Repeated and extensive BBB opening in the frontal lobe is safe and feasible, and results in decreased FBB SUVR and temporary improvement in related symptoms. As BBB opening is a safe procedure even for moderate-to-severe AD, in future studies it may be applied to AD patients without limitations on the disease severity.

## Supplementary Information

**Additional file 1**.** Table S1**: Inclusion and exclusion criteria.** Fig. S1**: Individual longitudinal changes in ^18^F-Florbetaben uptake after treatment.

## Data Availability

All data generated and/or analysed during the current study are not publicly available due to personal information of the patient, but available from the corresponding author on reasonable request.

## Abbreviations

AD: Alzheimer’s disease
Aβ: Amyloid-beta
BBB: Blood brain barrier
CGA-NPI: Caregiver-Administered Neuropsychiatric Inventory
CDR: Clinical Dementia Rating
CDR-SOB: CDR Sum of Boxes
COWAT: Controlled Oral Word Association Test
FBB: ^18^F-Florbetaben
FUS: Focused ultrasound
IADL: Instrumental Activities of Daily Living
MRI: Magnetic resonance imaging
MRgFUS: Magnetic resonance-guided FUS
MMSE: Mini-Mental State Examination
PET: Positron emission tomography
RCFT: Rey–Osterrieth Complex Figure Test
SUVR: Standardized uptake value ratio
