# Supplementary material for: Extensive frontal focused ultrasound mediated blood–brain barrier opening for the treatment of Alzheimer’s disease: a proof-of-concept study
Source: Transl Neurodegener. 2021 Nov 5;10:44. doi: 10.1186/s40035-021-00269-8 (PMC8570037; doi:10.1186/s40035-021-00269-8)
Supplement: Supplementary file 1 — Additional file 1. Table S1: Inclusion and exclusion criteria. Fig. S1: Individual longitudinal changes in 18F-Florbetaben uptake after treatment. [file 40035_2021_269_MOESM1_ESM.docx]

**Supplementary** **Table S1. Inclusion and exclusion criteria**

| Inclusion criteria |
| --- |
| Age between 50 and 85 years  Total score of 23 or less on the K-MMSE  Positive ^18^F-Florbetaben (FBB) PET scan  ^18^F-Fluorodeoxyglucose-PET scan suspected of Alzheimer disease-induced neurodegeneration  If being treated with medication approved for the treatment of AD, had been on stable dose for ≥3 months  Able to communicate during the ExAblate BBB opening procedure |
| Exclusion criteria |
| CGA-NPI severity score of more than 2 points for any of "Delusion", "Hallucination" or "Agitation/Aggression"  Known sensitivity/allergy or contraindications to MRI contrast agent (Gadovist®) or the ultrasound contrast agent (Definity®)  Contraindications to MRI such as non-MRI compatible implanted devices  MRI findings of active or chronic infection/inflammation, hemorrhage, tumor or space occupying lesion, meningeal enhancement, intracranial hypotension or severe ischemic changes  ≥30% of the skull area traversed by sonication is covered by scars, scalp disorder, or atrophic scalp  Active seizure disorder or epilepsy (seizures despite medical treatment)  History of a bleeding disorder, coagulopathy  Significant cardiac disease or unstable hemodynamics  Impaired renal function  Severe brain atrophy  Evidence of cranial or systemic infection  HIV positive, who have a high probability of developing HIV encephalitis due to the influx of HIV intto the brain parenchyma  A homozygosity of Apoliprotein E allele known to have a thin blood-brain barrier  Positive pregnancy test (women of childbearing potential)  A participant in other clinical trials or have experience in other clinical trials within 90 days from the screening date |

Abbreviations: CGA-NPI, Caregiver-Administrated Neuropsychiatry Inventory; K-MMSE, Korean version of Mini Mental State Exam; HIV, Human immunodeficiency virus

**
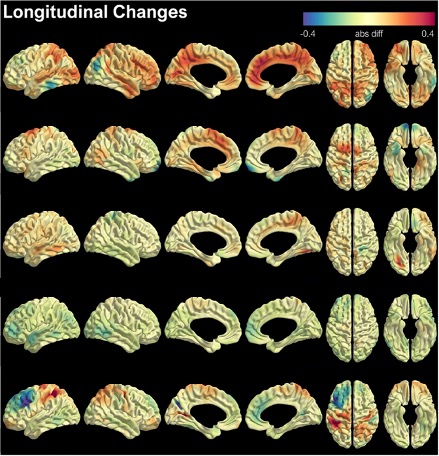
**

**Fig. S1. Individual longitudinal changes in ^18^F-Florbetaben uptake after treatment**

The individual changes in the ^18^F-Florbetaben SUVR after treatment of the five participants (each row is a single case, and case 1 to case 5 sequentially from top to bottom). The colors illustrate the absolute differences for the ^18^F-Florbetaben SUVR between pre- and post-treatment. SUVR, Standardized uptake value ratio
